# Application of vitamin E to antagonize SWCNTs-induced exacerbation of allergic asthma

**DOI:** 10.1038/srep04275

**Published:** 2014-03-04

**Authors:** Jinquan Li, Li Li, Hanqing Chen, Qing Chang, Xudong Liu, Yang Wu, Chenxi Wei, Rui Li, Joseph K. C. Kwan, King Lun Yeung, Zhuge Xi, Zhisong Lu, Xu Yang

**Affiliations:** 1 Section of Environmental Biomedicine, Hubei Key Laboratory of Genetic Regulation and Integrative Biology, College of Life Sciences, Central China Normal University, Wuhan 430079, P. R. China; 2 CAS Key Laboratory for Biomedical Effects of Nanomaterials and Nanosafety, CAS Key Laboratory of Nuclear Analytical Techniques, Institute of High Energy Physics, Chinese Academy of Sciences (CAS), Beijing 100049, P. R. China; 3 Deaparment of Chemical and Biomolecular Engineering, the Hong Kong University of Science and Technology, Clear Water Bay, Kowloon, Hong Kong, P. R. China; 4 Division of Environment, the Hong Kong University of Science and Technology, Clear Water Bay, Kowloon, Hong Kong, P. R. China; 5 Institute of Health and Environmental Medicine, Dali Road, Heping District, Tianjin 300050, P. R. China; 6 Institute for Clean Energy and Advanced Materials, Southwest University, Chongqing 400715, P. R. China; 7 These authors contributed equally to this work.

## Abstract

The aggravating effects of zero-dimensional, particle-shaped nanomaterials on allergic asthma have been previously investigated, but similar possible effects of one-dimensional shaped nanomaterials have not been reported. More importantly, there are no available means to counteract the adverse nanomaterial effects to allow for their safe use. In this study, an ovalbumin (OVA)-sensitized rat asthma model was established to investigate whether single walled carbon nanotubes (SWCNTs) aggravate allergic asthma. The results showed that SWCNTs in rats exacerbated OVA-induced allergic asthma and that this exacerbation was counteracted by concurrent administration vitamin E. A mechanism involving the elimination of reactive oxygen species, downregulation of Th2 responses, reduced Ig production, and the relief of allergic asthma symptoms was proposed to explain the antagonistic effects of vitamin E. This work could provide a universal strategy to effectively protect people with allergic asthma from SWCNTs or similar nanomaterial-induced aggravating effects.

Rapid advances in nanotechnology saw numerous engineered nanomaterials being fabricated. These were adopted for use in health care products, electronics, photonics, biomedicine, garments, sporting goods and clean energy, because of their unique and superior properties when compared to bulk materials. It is estimated that over 1300 manufacturer-identified nanomaterial-based products are publicly available and marketed; a number that increases by three or four per week^1^. Undoubtedly, widespread practical use of nanomaterials can provide many benefits in day-to-day life. However, their release into the environment might also pose potential threats to human health and the environment.

During the past decade, nanomaterial-induced negative effects on biological systems have been extensively reported^2^, and nanotoxicology, as an emerging subdiscipline of nanotechnology, has attracted tremendous research attention. As many nanomaterials are similar in size to cell organelles or biomolecules, they could penetrate into and interact with cells. Therefore, in early studies, most nanotoxicological data are obtained from *in vitro* cell culture systems^3^,^4^,^5^. In our previous investigations we found that quantum dots (QDs), silica nanoparticles, manganese dioxide nanowires, and single walled carbon nanotubes (SWCNTs) could induce cytotoxic effects in *Escherichia coli*, Scenedesmus obliquus, and *Hela* and rat aortic endothelial cells, respectively^6^,^7^,^8^,^9^. Although experimental conditions can be easily controlled in an *in vitro* cell culture system, data from these studies require verification with *in vivo* animal experiments. Thus, *in vivo* animal studies have recently been conducted to investigate the biodistribution of nanomaterials, including gold nanoparticles, QDs, SWCNTs, and graphene oxide nanosheets, in different organs^10^,^11^,^12^,^13^. Neurotoxicity and developmental toxicity as well as immunotoxicity induced by some typical nanomaterials have also been demonstrated^14^,^15^,^16^,^17^,^18^,^19^.

Allergic asthma, a common chronic inflammatory disease of the airway, and that requires continuous medical treatment, has become a heavy societal burden, particularly in developed countries. Environmental factors are regarded as important causes of allergic asthma. For example, during particulate matter (PM)-polluting events, asthmatic subjects show increases in respiratory symptoms, bronchoconstriction, medication use, bronchial hyperreactivity, and emergency care visits^20^. As most nanomaterials are in the size range of PM, exposure to nanomaterials might exacerbate allergic asthma; a hypothesis supported by several recently published articles. For example, titanium dioxide (TiO_2_), silica, and gold nanoparticles have been reported to aggravate pulmonary inflammation and airway hyper-responsiveness (AHR) in animal allergic asthma models^21^,^22^,^23^, implying that populations suffering from allergic asthma might possibly encounter great challenges from exposure to nanomaterials. The aggravating effects of nanomaterials on allergic asthma, including immuno-adjuvant effects on T helper (Th) 2-milieu, have been investigated^24^. However, there have been no studies of how to counteract or reduce the negative effects of nanomaterials and thus facilitate their safe use.

In this study, an animal allergic asthma model involving lung function tests was first established and used to explore the aggravating effects of SWCNTs, a typical one dimensional (1-D) nanomaterial that has been widely used in practical applications. This model allows the examination of allergic asthma symptoms by assessing cytokine and serum immune globulin concentrations, airway leukocyte infiltration, histopathological changes of lung tissue, and AHR. This system was then used to examine reactive oxygen species (ROS) accumulation involved in these aggravating effects, ROS elimination effects by vitamin E (Ve), and finally the antagonistic effects of Ve on SWCNTs-induced immuno-adjuvant effects. This study has the potential to provide an effective strategy for protecting people with allergic asthma from asthma aggravation caused by nanomaterials.

## Results

### Characterization of SWCNTs

Since nanomaterial induced biological responses are closely related to their properties, SWCNTs were initially characterized by scanning and transmission electron microscopies (SEM and TEM, respectively) and by Raman spectroscopy. Similar to previous reports^25^, SWCNTs bundle structures were observed in distilled water rather than the individual dispersed tubes even after 30 min of sonication (Fig. S1A). Tween-80, an effective biocompatible surfactant, was used to obtain well-dispersed SWCNTs by creating electrostatic repulsion forces among the nanotubes, thus increasing SWCNTs' solubility^26^,^27^. A solution of 0.05% (by vol) aqueous Tween-80 solution produced individual, dispersed SWCNTs (Fig. S1B) and was thus used to disperse SWCNTs in subsequent experiments. TEM showed the single-walled tube structure of this material as well as the negligible presence of amorphous carbon and metal catalyst particles (Fig. S1C). SWCNTs' structure and purity were further analyzed by Raman scattering analysis, which exhibited well-defined G and D-bands (1593.09 and 1337.59 cm^−1^, respectively), with a G/D ratio of 13.64, suggesting that these SWCNTs were relatively free of defects and impurities (Fig. S1D). Raman radial breathing mode (RBM) peak analysis revealed the range of SWCNT diameters to be from 0.93 to 1.63 nm. These characterization data indicated that the quality of these SWCNTs perfectly met the requirements of the following experiments.

### Effects of SWCNTs on lung cytokine and serum Ig concentrations in the presence of OVA and the elimination effects of Ve

**Figure 1:**
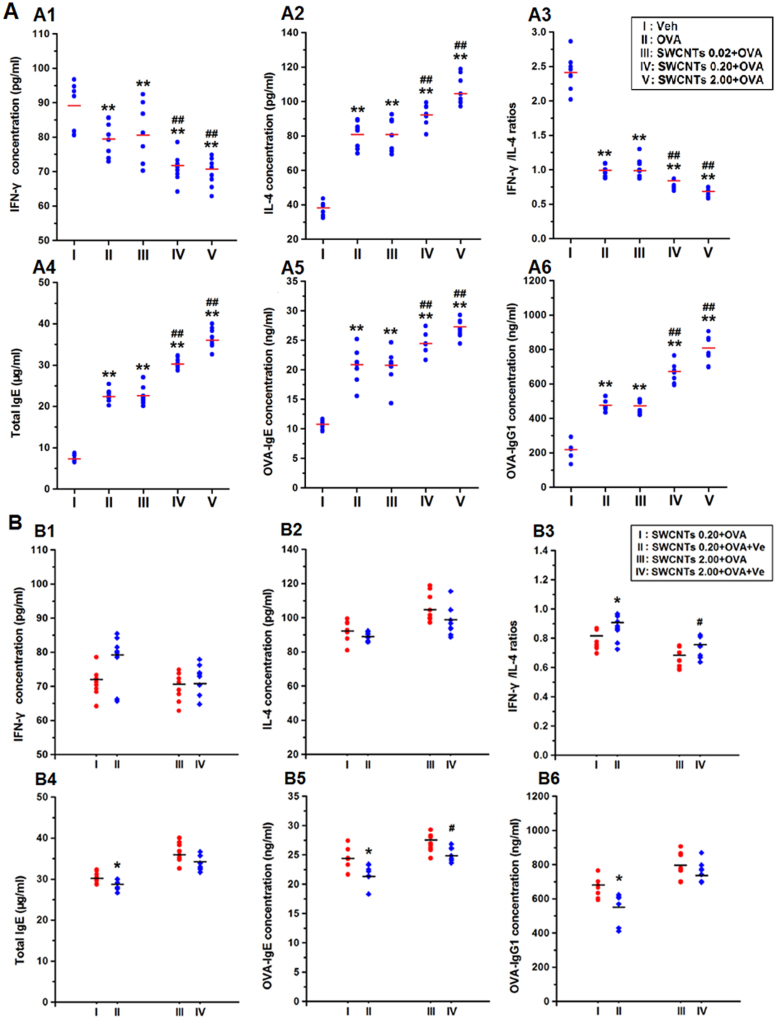
Effects of SWCNTs on lung cytokine and serum Ig concentrations in the presence of OVA and the elimination effects of Ve. (A1–A3), concentrations of IFN-γ, IL-4, and IFN-γ/IL-4 ratios, respectively, in lung with OVA; (A4–A6), concentrations of total IgE, OVA-sIgE, and OVA-sIgG1, respectively, in serum with OVA; **, *p* < 0.01, compared with vehicle group; and ##, *p* < 0.01, compared with OVA group. (B1–B3), IFN-γ, IL-4, IFN-γ/IL-4 ratios, with protective effects of Ve in the presence of combined OVA + SWCNTs treatment; (B4–B6), tIg E, OVA-sIgE, and OVA-sIgG1, respectively, with protective effects of Ve in the presence of combined OVA + SWCNTs treatment; *, *p* < 0.05, compared with SWCNTs 0.20 + OVA group; and #, *p* < 0.05, compared with SWCNTs 2.00 + OVA group.

Total-immunoglobulin (Ig) E (tIgE), specific IgE (sIgE), and specific IgG1 (sIgG1) together with Interferon-γ (INF-γ, a Th1 cytokine) and interleukin-4 (IL-4, a Th2 cytokine) were assayed to verify at the molecular level the establishment of a rat asthma model. All ovalbumin (OVA)-sensitized groups displayed decreases in IFN-γ concentrations and increases in IL-4 concentrations compared with the vehicle control group (Fig. 1A1 and 1A2). The ratios of IFN-γ to IL-4 were also lower (Fig. 1A3), suggesting that the Th1/Th2 balance was broken and that the Th2 response played a dominant role in OVA-treated rats. In agreement with the findings of the cytokine assays, after OVA sensitization, there are significant increases in tIgE, OVA-sIgE, and OVA-sIgG1 concentrations (Fig. 1A4–A6), which matched the Ig indicators of the rat allergic asthma model very well. More interesting is the observation that 0.2 and 2.0 mg/kg SWCNTs treatment facilitated Th2 immune responses in a dose-dependent manner (Fig. 1A1–A3); productions of tIgE, OVA-sIgE, and OVA-sIgG1 were also facilitated after 0.2 and 2 mg/kg SWCNTs treatment in the presence of OVA (Fig. 1A4–A6). Examination of the effects of 0.02 mg/kg SWCNTs on lung cytokine and serum Ig concentrations in the presence of OVA revealed no significant changes compared with the OVA groups. Interestingly, treatment with vitamin E could reduce the imbalance of Th1 and Th2 immune response induced by SWCNTs (Fig. 1B3). Furthermore, OVA-combined with 0.2 mg/kg SWCNTs and vitamin E protect group decreased the level of tIgE, OVA-sIgE, and OVA-sIgG1 compared with the SWCNTs 0.2 + OVA group (Fig. 1B4–B6).

### Effects of SWCNTs on inflammatory cell recruitment in the presence of OVA and the elimination effects of Ve

**Figure 2:**
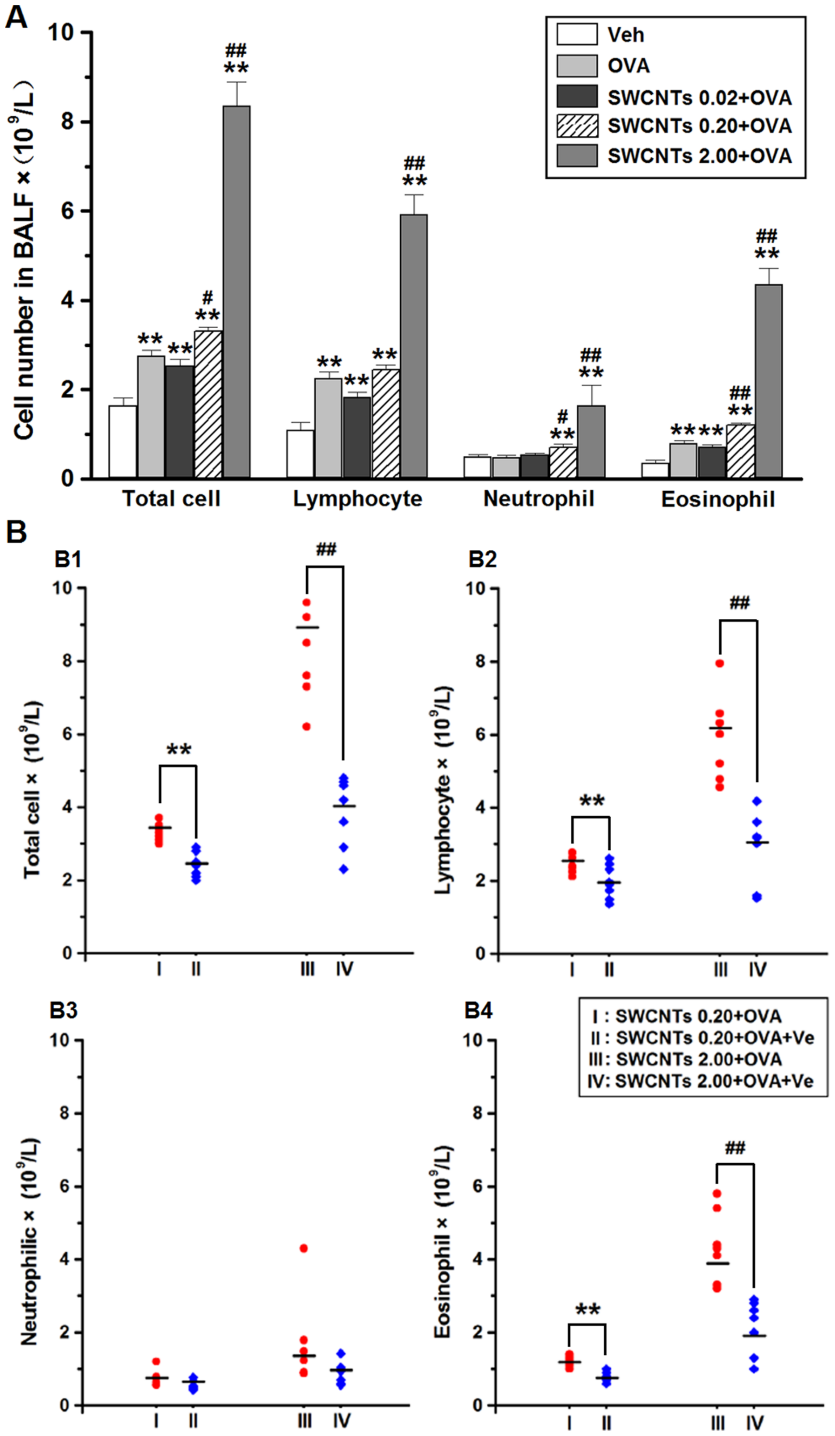
Effects of SWCNTs on inflammatory cell recruitment in the presence of OVA and the elimination effects of Ve. (A), effects of SWCNTs on inflammatory cell recruitment in the presence of OVA; **, *p* < 0.01, compared with vehicle group; and #, *p* < 0.05 and ##, *p* < 0.01, compared with OVA group. (B1–B4), total cells, lymphocytes, neutrophils, and eosinophils, respectively, protective effects of Ve on inflammatory cell recruitment in the presence of combined OVA + SWCNTs treatment; **, *p* < 0.01, compared with SWCNTs 0.20 + OVA group; and ##, *p* < 0.01, compared with SWCNTs 2.00 + OVA group.

In this study, the exploration of the cellular profile of bronchoalveolar lavage fluid (BALF) showed that the number of total cells, lymphocytes, and eosinophils in the OVA-treated group were greater than those in the vehicle only group (Fig. 2A). Combined treatment with OVA + SWCNTs further increased the number of total cells and of these two cell types in addition to neutrophils. In addition to influences on Th2 responses and Ig concentrations, Ve also acted at the cellular level to relieve the aggravating effects of SWCNTs. Oral administration of Ve reduced the number of inflamed cells that were due to the presence of SWCNTs and OVA (Fig. 2B1–B4). The number of total cells, lymphocytes, and eosinophils in the SWCNTs 0.20 + OVA + Ve and SWCNTs 0.20 + OVA + Ve groups were much smaller than those in the same treatment groups without Ve addition, indicating Ve greatly attenuated airway inflammation.

### SWCNTs treatment effects visualized as histopathological lung changes in the presence of OVA and the elimination effects of Ve

**Figure 3:**
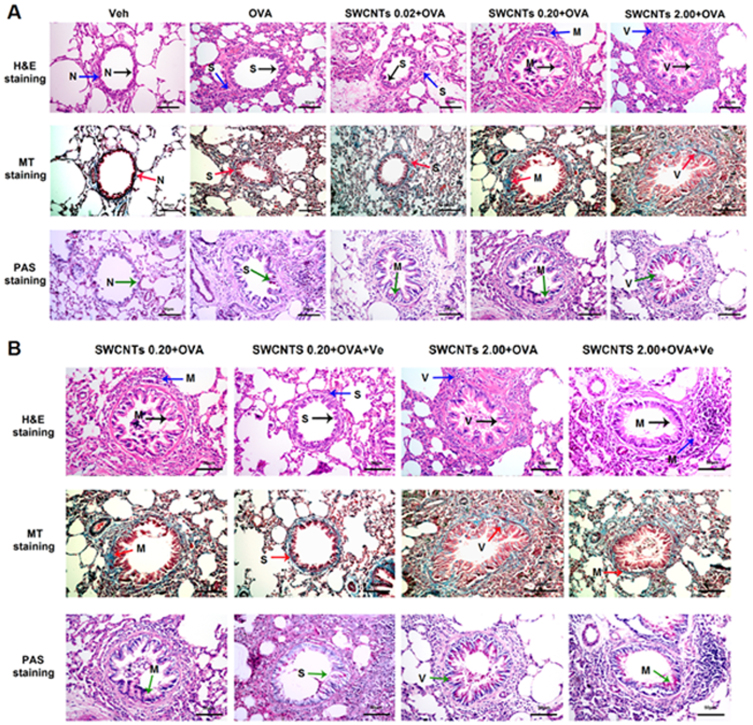
Histopathological lung changes. (A), SWCNTs treatment effects visualized as histopathological lung changes in the presence of OVA. (B), protective effects of Ve visualized as histopathological changes in lung tissue caused by combined OVA + SWCNTs treatments. Black arrow, bronchial remodeling; blue arrow, lung tissue cell infiltration; red arrow, subepithelial collagen deposition (blue colored stain); green arrow, mucus hypersecretion (pale pink colored stain); N, S, M, and V indicate normal, slight, medium, and severe changes, respectively.

In this study we used three staining methods, including hematoxylin and eosin (H&E), Masson's trichrome (MT), and periodic acid-Schiff (PAS) staining, to reveal changes in pulmonary histology. H&E staining is a classical staining method for visualizing airway remodeling, revealing typical pathological features of asthmatic airway inflammation and structural alterations, including leukocyte infiltration in surrounding peribronchiolar areas, epithelial folding, and thickened subepithelial cell layers. MT staining discloses peribronchial collagen deposition, and PAS staining exposes increased mucus production that occurs after allergen sensitization and challenge due to goblet cell hyperplasia. Combined treatments of SWCNTs + OVA appeared to worsen histological changes in lung tissues compared with the OVA group. Moreover, the higher the SWCNTs concentration, the more severe the pathological changes (Fig. 3A). Airway remodeling observed in these experiments provided further evidence for the feasibility of this asthma animal model in showing the exacerbating effects of SWCNTs. It also showed that treatment with vitamin E in OVA + SWCNTs 0.2 and OVA + SWCNTs 2 groups markedly reduced the degree of infiltration of inflammatory cells, mucus overproduction, fibrosis and goblet-cell hyperplasia (Fig. 3B).

### Effects of SWCNTs treatment on AHR in rats in the presence of OVA and the elimination effects of Ve

**Figure 4:**
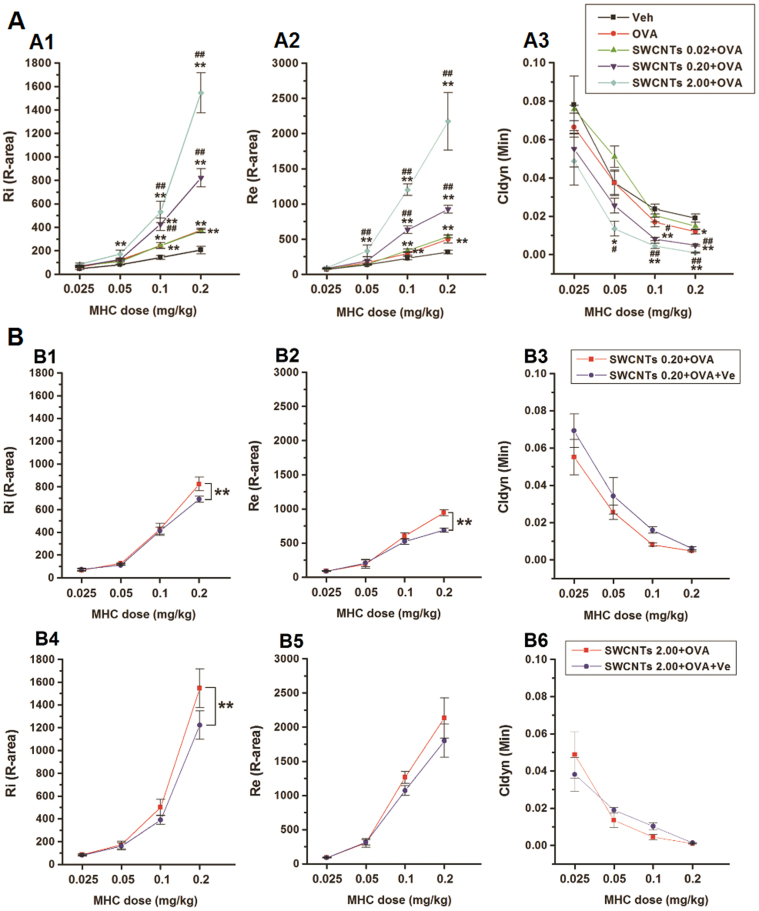
Effects of SWCNTs treatment on AHR in rats. (A1–A3), Ri, Re, and Cldyn values, respectively, of vehicle, OVA, and OVA + SWCNTs groups; **, *p* < 0.01, compared with vehicle group; and *p* < 0.01, compared with OVA group. (B1–B3) represent Ri, Re, and Cldyn values, respectively, of SWCNTs 0.20 + OVA, and SWCNTs 0.20 + OVA + Ve groups; and **, *p* < 0.01, compared with SWCNTs 0.20 + OVA group. (B4–B6) represent Ri, Re, and Cldyn values, respectively of SWCNTs 2.00 + OVA, and SWCNTs 2.00 + OVA + Ve groups, respectively; and **, *p* < 0.01, compared with SWCNTs 2.00 + OVA group.

OVA treatment followed by a methacholine (MCH) challenge assay produced an increase in the R-areas of respiratory resistances (Re and Ri, respectively) and a decrease in dynamic lung compliance (Cldyn) (Fig. 4A1–A3), which supported the authenticity of this allergic asthma model. Continuous upward shifts of the Ri and Re curves and downward shifts of the Cldyn curves were detected as the SWCNT concentration increased from 0.02 to 2.0 mg/kg in the presence of OVA(Fig. 4A1–A3), suggesting that SWCNTs adversely affected both large and small airways of the lung. Treatment with vitamin E dramatically reduced Ri and Re results in the OVA + SWCNTs 0.2 and OVA + SWCNTs 2 groups, respectively (Fig. 4B1–B6).

### SWCNTs effects on oxidative stress in the presence of OVA and the ROS elimination effects of Ve

**Figure 5:**
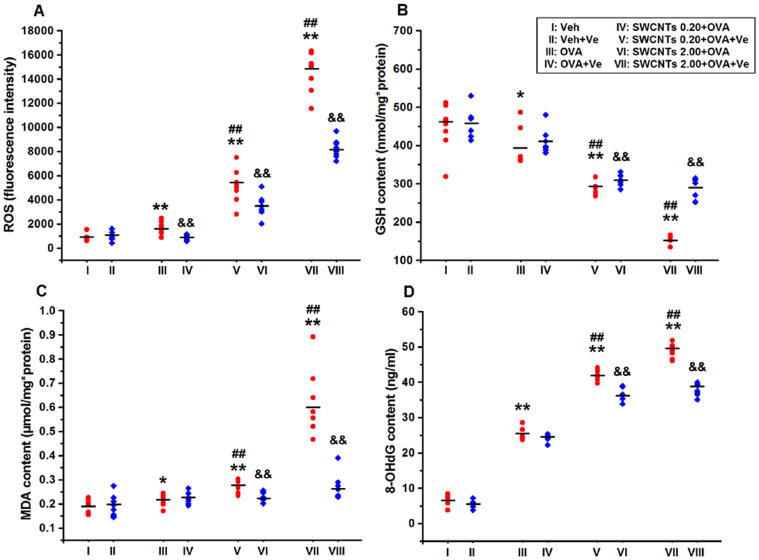
SWCNT effects on oxidative stress in the presence of OVA and the ROS elimination effects of Ve. Concentrations of ROS, GSH, MDA, and 8-OHdG, (A–D), respectively, in the lung; *, *p* < 0.05; **, *p* < 0.01, compared with vehicle group; ##, *p* < 0.01, compared with OVA group; and &&, *p* < 0.01, compared with Ve-untreated group with same SWCNT concentration.

To estimate the level of oxidative stress in the lung induced by SWCNTs, we measured the reactive oxygen species (ROS), glutathione (GSH), malondialdehyde (MDA), and 8-hydroxy-2′-deoxyguanosine (8-OHdG) concentrations. In this study we found that, co-exposure with OVA, 0.2 and 2 mg/kg SWCNTs exposure groups have the ability to increase oxidative stress-related biomarkers such as ROS, MDA and 8-OHdG in the lung tissue compared with vehicle and OVA group (Fig. 5A, C and D). In addition, as ROS levels increased, the GSH content was depleted in these two groups (Fig. 5B). This study also showed that oral administration of vitamin E after instillation could inhibit the oxidative stress induced by SWCNTs (Fig. 5A–D).

## Discussion

**Figure 6:**
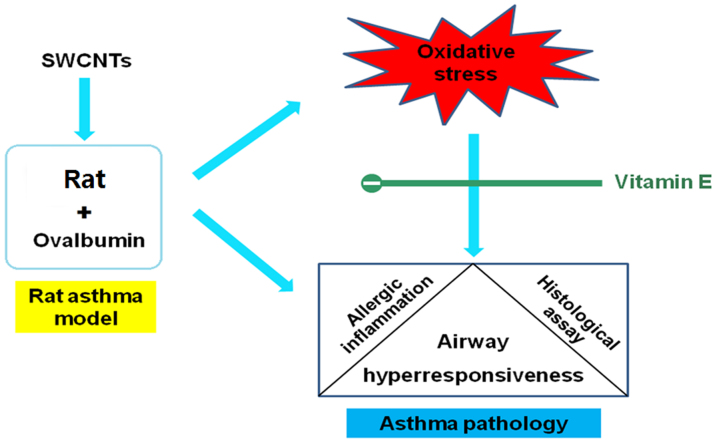
Potential mechanism of SWCNTs-induced exacerbation of allergic asthma (Acknowledge: Figure 6 was made by Mr. Jinquan Li, one of our co-authors).

In this study we confirmed that SWCNTs administered intratracheally exacerbate allergen-related airway inflammation with airway remodeling in rats. AHR, which is a critical clinical hallmark of asthma, was also found to show the deterioration induced by SWCNTs. More importantly, by using vitamin E, we demonstrated that oxidative stress is an important mechanism of SWCNTs-induced aggravating effects (Fig. 6). This work could provide a universal strategy for the effective protection of people with allergic asthma from SWCNTs or similar nanomaterial-induced aggravation.

TIgE, sIgE, and sIgG1 concentrations, regulated by Th1 and Th2 immune responses, are important in the clinical diagnosis of allergic asthma^28^,^29^,^30^,^31^. Inflammatory cell recruitment in the airway is a key event in the pathological progress of asthma^32^,^33^. It is worth mentioning that increased eosinophils found in airway secretions is a fundamental feature of asthma and correlates with asthma severity^34^,^35^,^36^. Airway remodeling, the consequence of airway inflammation, is a well-established feature of asthma^37^,^38^,^39^. AHR is another critical, clinical hallmark of allergic asthma^29^. Rat AHR has been evaluated in terms of expiratory and inspiratory resistances and Cldyn using MCH challenge tests. The R-areas of the respiratory resistances and the change in Cldyn represent variations in the large airways and the state of small airways, respectively^40^,^41^. In this study, Lung cytokines and serum Ig concentrations as well as the BALF cell profile indicated that airway inflammation developed after OVA treatment and that 0.2 and 2.0 mg/kg of SWCNTs further aggravated OVA-induced allergic inflammation. The exacerbating effects of SWCNTs/MWCNTs on airway allergic inflammation have been verified in a number of previous works^17^,^18^,^24^,^39^,^42^,^43^,^44^. The results in the present study agree well with those reported in an investigation that examined the effects of a single SWCNT's concentration (50 μg/animal)^24^.

Based on the results in this study, the successful establishment of a rat allergic asthma model was demonstrated. The aggravating effects of SWCNTs on OVA-induced allergic asthma were also verified by detecting cytokine and serum Ig concentrations, airway leukocyte infiltration, histopathological changes of lung tissue and AHR. Considering the rapid development of nanotechnology, more nanomaterials can be expected to enter the environment and the risk associated with nanomaterial exposure will greatly increase for people with allergic asthma. Therefore, there is a great demand for a strategy for safe nanomaterial use.

SWCNTs' ability to induce nanotoxicity *via* a ROS-dependent pathway has been well documented in the existing literature^42^,^43^,^44^. Both SWCNTs and MWCNTs could induce cell apoptosis even though minor morphological differences were observed, indicating that SWCNT and MWCNT may in general affect the lung homeostasis^5^. Here, the effects of SWCNTs on pulmonary oxidative stress in the presence of OVA were assessed and it was found that OVA treatment led to increases in ROS, MDA, and 8-OHdG content as well as a decrease of GSH content, indicating the production of oxidative stress. Similar to the data from the above asthma model assays, 0.2 and 2.0 mg/kg of SWCNTs exposure combined with OVA sensitization clearly exacerbated SWCNTs impact, resulting in severe oxidative stress (Fig. 5). Notably, ROS concentration, in particular the GSH concentration is closely related to Th1/Th2 responses^45^. A recent report suggests that ROS plays an important role in the exacerbating effects of SWCNTs on airway allergic inflammation^24^. Thus, the ROS pathway could be one possible mechanism for SWCNT aggravation on OVA-induced allergic asthma. In this study, Ve, an antioxidative reagent, was shown to effectively relieve SWCNT-caused oxidative stress in the presence of OVA (Fig. 5). Actually, the importance of Ve has been demonstrated in a previous work, which observed the enhanced pulmonary inflammatory response and oxidative stress induced by SWCNTs in mice fed with a Ve-deficient diet^46^. Based on those findings^46^,^47^, it is speculated that Ve might possibly be used to antagonize immuno-adjuvant effects of SWCNTs in this rat allergic asthma model.

In order to confirm the proposed hypothesis, we investigated the effects of Ve on cytokine and serum Ig concentrations, airway leukocyte infiltration, histopathological changes, and AHR in the presence of a combined treatment with SWCNTs + OVA. Although the addition of Ve did not alter the IFN-γ and IL-4 concentrations significantly, it did cause increased IFN-γ/IL-4 ratios in both the SWCNTs 0.2 + OVA + Ve and SWCNTs 2.0 + OVA + Ve groups (Fig. 1B3). Observed decreases in tIgE, OVA-sIgE, and OVA-sIgG1 concentrations further confirmed the protective effects of Ve on OVA + SWCNTs-caused allergic inflammation. Ve showed no effect on OVA-caused enhancement of cytokine and Ig concentrations, it is suggest that this agent might target SWCNTs-induced responses (Fig. S2), and Ve induced decrease in serum Ig production in combined SWCNTs + OVA groups via regulating the Th2 responses. As reported in the existing literature^39^,^48^, airway neutrophilia and fibrosis were discovered in SWCNT-treated groups. SWCNT-induced increase in production of platelet-derived growth factor (PDGF) and transforming growth factor (TGF)-β1, two growth factors that mediate the pathogenesis of fibrosis, may be possible reasons for these observations^39^.

As airway remodeling is thought to be the consequent event of chronic airway inflammation, it was necessary to inspect Ve's effect at the tissue level. In comparison to the OVA + SWCNTs groups without Ve, the degrees of bronchial remodeling, pulmonary infiltration, subepithelial collagen deposition, and mucus hypersecretion were less in the SWCNTs 0.20 + OVA + Ve and SWCNTs 0.20 + OVA + Ve groups. Data from tissue level examinations also supported Ve's protective abilities against the aggravating effects of SWCNTs on allergic asthma (Fig. 3B). AHR is one of the defining features of asthma. Moreover, asthma symptoms, such as coughing, wheezing, chest tightness, and shortness of breath are based on the presence of AHR. Therefore, AHR assays must be conducted to verify whether Ve functions by attenuation of SWCNTs-caused asthma exacerbation effects. In all Ve-treated groups, downwards shifts in the Ri and Re curves were observed as well as upward shifts of the Cldyn curves, providing more strong evidence of Ve's protective effects (Fig. 4).

The use of 100 mg Ve/kg on the vehicle control and OVA-sensitized did not have to any adverse effects on animals (Fig. S2–S5). Thus, Ve was concluded to be useful as a protective reagent for attenuating SWCNTs aggravation on OVA-induced allergic asthma. We propose the following mechanism to explain this effect. First, Ve eliminates ROS caused by exposure to SWCNTs, reducing the oxidative stress in the pulmonary cells. Next, GSH recovers to a higher concentration, causing downregulation of the Th2 response and decreasing serum Ig concentrations. This in turn reduces the number of inflamed cells in the lung and hinders the airway remodeling process which finally leads to relief of allergic asthma symptoms.

In this study, an OVA-sensitized rat asthma model was established for the purpose of investigating the aggravation of allergic asthma by SWCNTs. By detecting changes of asthma-related cytokine and serum Ig concentrations, lung immune cellular profiles, histopathological changes of lung tissue, and AHR, this OVA-sensitized rat asthma model was successfully demonstrated. SWCNTs' exacerbation of OVA-induced allergic asthma was also verified at SWCNT concentrations of 0.2 and 2.0 mg/kg. These findings indicated that not only 0-D, particle-shaped nanomaterials but also 1-D, tube-shaped nanomaterials could exacerbate allergic asthma. Subsequently, Ve, an antioxidative reagent, was introduced to antagonize SWCNTs-induced immuno-adjuvant effects via oxidative stress reductions. The effective antagonistic effects of Ve on SWCNTs effects were confirmed by comparing asthma symptoms of OVA + SWCNTs and OVA + SWCNTs + Ve groups. Based on these results, the mechanism for Ve's antagonistic effects was proposed, involving ROS elimination, downregulation of Th2 responses, reduced Ig production, and relief of allergic asthma symptoms. This study suggested that Ve supplements might protect people with allergic asthma from SWCNTs or other nanomaterial-caused aggravating effects. As Ve has been previously reported to reduce nanotoxicity via ROS elimination, this study might provide support for a universal strategy for the safe, practical use of nanomaterials.

## Methods

### Animals

Male Wistar rats (6–7 weeks old) were purchased from the Hubei Province Experimental Animal Center (Wuhan, China). All animal experiments were conducted in accordance with National Institutes of Health Guide for the Care and Use of Laboratory Animals, and were approved by the Office of Scientific Research Management of Central China Normal University (March 1, 2012 CCNU-IACUC-2012-011).

### Main reagents and kits

All reagents were from Sigma unless otherwise indicated. Rat enzyme-linked immunosorbent assay (ELISA) kits for 8-OHdG and total IgE were purchased from Kamiya Biomedical Company (Seattle, WA, USA). Rat ELISA kits for OVA-lgG1 and OVA-IgE were purchased from Boster Bio-engineering Co., Inc. (Wuhan, China). Rat ELISA kits for IL-4 and IFN-γ were bought from R&D Systems, Inc. (Minneapolis, MN, USA).

### SWCNTs and physicochemical characterization

SWCNTs synthesized by the chemical vapor deposition method (Sigma-Aldrich, Inc.) were used in this study. Their physical and chemical properties were characterized by several techniques, including Raman spectroscopy, SEM, and TEM. Raman spectra were collected using a Raman microscope (Renishaw inVia Plus, Renishaw Inc., Hoffman Estates, IL, USA) with a 50× air objective lens and a 633 nm (1.96 eV) laser. Diameters and shapes of SWCNTs were characterized using SEM and TEM (JEOL-6700F and JEOL JEM-2010, respectively, JEOL Ltd., Tokyo, Japan).

### Experimental protocol

SWCNTs were dispersed in Tween-80 solution (0.05% Tween-80 in distilled water) and the suspensions sonicated in a 12–15°C sonicator water bath (KQ-100 DE, Ultrasonic Instrument Co. Ltd., Kunshan, China) for 15 min. This well dispersed SWCNT suspension was used as a stock solution for further uses. Exposure solutions were freshly prepared by diluting the stock solution with distilled water and sonication for another 15 min before instillation. After such processing, exposure solutions were stable for at least 24 h.

Seventy-two male Wistar rats were randomly divided into nine exposure groups of eight rats per group, comprising (1) a vehicle group (Veh), (2) OVA-sensitized group (OVA), (3) OVA-combined with 0.02 mg SWCNTs/kg body weight (bw) group (SWCNTs 0.02 + OVA), (4) OVA-combined with 0.20 mg SWCNTs/kg bw group (SWCNTs 0.20 + OVA), (5) OVA-combined with 2.00 mg SWCNTs/kg bw group (SWCNTs 2.00 + OVA), (6) vehicle and Ve-protected (100 mg Ve/kg) group (Veh + Ve), (7) OVA-sensitized and Ve-protected group (OVA + Ve), (8) OVA-combined with 0.20 mg SWCNTs/kg bw and Ve-protected group (SWCNTs 0.20 + OVA + Ve), (9) OVA-combined with 2.00 mg SWCNTs/kg bw and Ve-protected group (SWCNTs 2.00 + OVA + Ve). The details of the protocol are shown in Figure S10, Supporting Information.

The rats were instilled intratracheally^22^ with 0.1 mL of three different SWCNT suspensions or distilled water containing 0.05% Tween 80 once every three days, for a total of 13 instillations, and sensitized with OVA + Al(OH)_3_ (200 mg OVA and 6.5 mg Al(OH)_3_ in 1 mL saline each time) or saline (1 mL saline each time) by subcutaneous injection on day 6, 20, and 27. This was then followed by an aerosol challenge of 1% OVA (30 min/d) from days 33 to 39 (7 times) using an ultrasonic nebulizer (Yuyue 402A type I, Shanghai Yu Yue Medical Equipment Co., Inc., Shanghai, China). Oral administration of Ve (100 mg/kg) was given 3 h after administration of SWCNTs (0.20 or 2.00 mg/kg bw) as an antioxidant in OVA-treated groups.

Adverse effects in assessing concentrations of oxidative stress-related biomarkers, Th cytokine and Ig concentrations, due to the influence of methacholine used in AHR assessments were avoided by performing each experiment in duplicate. In the first round, after treatment the 72 rats were used directly for measuring Th cytokine concentrations and oxidative stress-related biomarkers in lung homogenates and serum Ig production. In the second round, the 72 rats were treated with the same protocol and then utilized in AHR tests, BALF sample collections, and lung histological assays.

### Preparation of serum and lung tissue homogenate

24 h after the final challenge, the rats were anesthetized intraperitoneally with pentobarbital sodium (100 mg/kg bw) in the first round of experiments. Serum samples were then collected from heart blood by centrifugation (3000 rpm at 24°C for 15 min) and stored at −70°C.

After serum collection, the rats were sacrificed by cervical dislocation and the whole lung removed by medical scissors and rinsed in ice-cold PBS. Next, lung tissue was homogenized in a glass homogenizer on ice, using 10 mL/g of ice-cold PBS at pH 7.5 to produce a 10% tissue homogenate. Half of the homogenate was centrifuged at 10,000 rpm and 4°C for 10 min and the supernatant collected and frozen at −70°C for later assessment of the ROS, GSH, and MDA. The remaining homogenate was used to test for 8-OH-dG, IL-4, and IFN-γ, according to manufacturer protocols.

### Quantitative analyses of lung protein concentrations of Th cytokines and serum Ig

The lung protein levels of IL-4 and IFN-γ, and serum concentrations of T-IgE, OVA-sIgE, and OVA-sIgG1 were measured using ELISA kits, according to manufacturer protocols. Sensitivities of the ELISA kits were 5 pg/mL for IL-4, 10 pg/mL for IFN-γ, 1 ng/mL for T-IgE, OVA-sIgG1 and 0.1 ng/mL for OVA-sIgE.

### ROS assay

ROS concentrations were determined based on the reactions between ROS and 2′,7′-dichlorofluorescein diacetate (DCFH-DA). After transfer into cells, DCFH-DA is cleaved to form a highly fluorescent compound, DCFH, which is transformed into highly fluorescent DCF upon reaction with ROS. DCF can be quantified by a fluorescence reader (FLx 800 Multi-Detection Microplate Reader, BioTek Instruments, Wisnooski, VT, USA). Supernatant of lung tissue homogenate was diluted 2-fold in PBS, and 100 μL of diluted supernatant mixed with 100 μL DCFH-DA (diluted 1000-fold with 10 μM dimethyl sulfoxide, DMSO) and placed in the wells of a 96-well microplate. The reaction mixture was allowed to sit in complete darkness at 37°C for 5 min and the ROS concentration then evaluated using a fluorescence reader with 485 and 520 nm for excitation and emission, respectively^49^.

### GSH assay

Thiols, such as GSH, can react with 5,5′-dithio-bis-(2-nitrobenzoic) acid (DTNB) in the dark to form yellow compounds^50^. A sample of 200 μL of lung tissue homogenate supernatant was mixed with 50 μl of 10% trichloroacetic acid solution (TCA) to precipitate all proteins present. The sample was then centrifuged at 10,000 rpm at 4°C for 10 min to produce a clear supernatant. The pH was adjusted to 7.5 to yield a color-change reaction with DTNB. A 50 μL volume of this sample was next mixed with 150 μL DTNB (60 μg/mL, diluted 50-fold from DMSO-dissolved stock solution), transferred to a well in a 96-well plate, and placed in the dark at room temperature for 5 min. Finally, the absorbance in each well was measured at 412 nm. The total GSH (nmol/L) in each sample was then estimated using the equation OD412/0.0023, an extrapolation from Beer's Law. The protein concentration was determined using the Lowry assay^51^.

### MDA assay

MDA concentration in rat lung tissue homogenate was measured using a previously described procedure^52^. The protein concentration was determined using the Lowry assay.

### 8-OH-dG ELISA assay

The 8-OH-dG concentration in the lung supernatants was measured using an ELISA kit, according to manufacturer instructions. The sensitivity of this ELISA kit was 0.5 ng/mL.

### Lung function measurement

In the second round of experiments, 24 h after the final aerosol challenge, the 72 rats were tested for AHR, using the AniRes2005 lung function system (Bestlab, version 2.0, China), according to the manufacturer instructions. Ri and Re R-areas, the graphic area between the peak value and baseline, and the valley value of Cldyn^40^ were recorded for further analysis.

### BAL and cell counting

After measuring AHR, the lungs of each rat were lavaged in situ with 1-mL volumes of saline instilled by syringe. The rat's chest was gently massaged and rinsed for ~1 min, and then the liquid withdrawn with the same syringe, with the process repeated 3 times, and the total lavage fluid collected and combined. The recovery rates of different rats were ≥90%. Then, samples of bronchoalveolar lavage fluid were centrifuged at 1,500 rpm at 4°C for 10 min and the cell sediment suspended in saline to facilitate cell counting. Total cells, neutrophils, eosinophils, and lymphocytes in suspension were counted using a Blood Cell Analysis system (MTN-21, Matee3nu Technology Corp., Jinan, China).

### Lung histological assay

After collecting bronchoalveolar lavage fluid, the left lung of a rat was removed for histopathology slice preparation. All samples were fixed in 10% formalin solution for 24 h at room temperature, cut into pieces, and separate pieces stained with H&E, MT, and PAS, as previously described^53^,^54^. Stained pieces were embedded in paraffin, sectioned into 10-μm slices, and observed using the DM 4000B microscope (Leica Microsystems GmbH, Wetzlar, Germany).

### Statistical analysis

AHR assessment data and cell counting data are presented as the mean ± standard error of the mean. Other data are presented as the mean ± scatter. Statistical graphs were generated using Origin 8.0 Software (OriginLab Corp., Berkeley, CA, USA). One-way ANOVA combined with Fisher's protected t-test was used to determine the significance of the differences between groups. Significance values of *p* < 0.05 were considered significant and *p* < 0.01 considered extremely significant.

## Author Contributions

L.J., L.L., L.Z., Y.X., K.J. and Y.K. designed the experiments. L.J., L.L., C.H. and C.Q. performed the experiments. L.J., L.L. and Y.X. analyzed the data. L.X., W.Y., W.C., L.R. and X.Z. contributed reagents/materials/analysis tools/funding. L.J., L.L., L.Z., Y.X., K.J. and Y.K. took part in the preparation and revision of the manuscript. All authors have given approval to the final version of the manuscript.

## Supplementary Material

Supplementary Information Supporting_Information
