# Supplementary material for: Application of vitamin E to antagonize SWCNTs-induced exacerbation of allergic asthma
Source: Sci Rep. 2014 Mar 4;4:4275. doi: 10.1038/srep04275 (PMC3940970; doi:10.1038/srep04275)
Supplement: Supplementary Information — Supporting_Information [file srep04275-s1.pdf]

Supporting Information

Application of vitamin E to antagonize SWCNTs-induced exacerbation of allergic asthma

Jinquan Li[#1], Li Li [#1], Hanqing Chen[2], Qing Chang[3], Xudong Liu[1],Yang Wu[1], Chenxi Wei [1], Rui Li[1], Joseph K C Kwan[4], King Lun Yeung [3,4], Zhuge Xi [5], Zhisong Lu[* 6] and Xu Yang[* 1]

[1]Section of Environmental Biomedicine, Hubei Key Laboratory of Genetic Regulation and Integrative Biology, College of Life Sciences, Central China Normal University, Wuhan 430079, P. R. China. [2]CAS Key Laboratory for Biomedical Effects of Nanomaterials and Nanosafety, CAS Key Laboratory of Nuclear Analytical Techniques, Institute of High Energy Physics, Chinese Academy of Sciences (CAS), Beijing 100049, P. R. China. [3]Deaparment of Chemical and Biomolecular Engineering and [4]Division of Environment, the Hong Kong University of Science and Technology, Clear Water Bay, Kowloon, Hong Kong, P. R. China. [5]Institute of Health and Environmental Medicine, Dali Road, Heping District, Tianjin 300050, P. R. China. [6]Institute for Clean Energy and Advanced Materials, Southwest University, Chongqing 400715, P. R. China.

*Correspondence to Xu Yang (Email): yangxu@mail.ccnu.edu.cn; Zhisong Lu, (Email): zslu@swu.edu.cn.

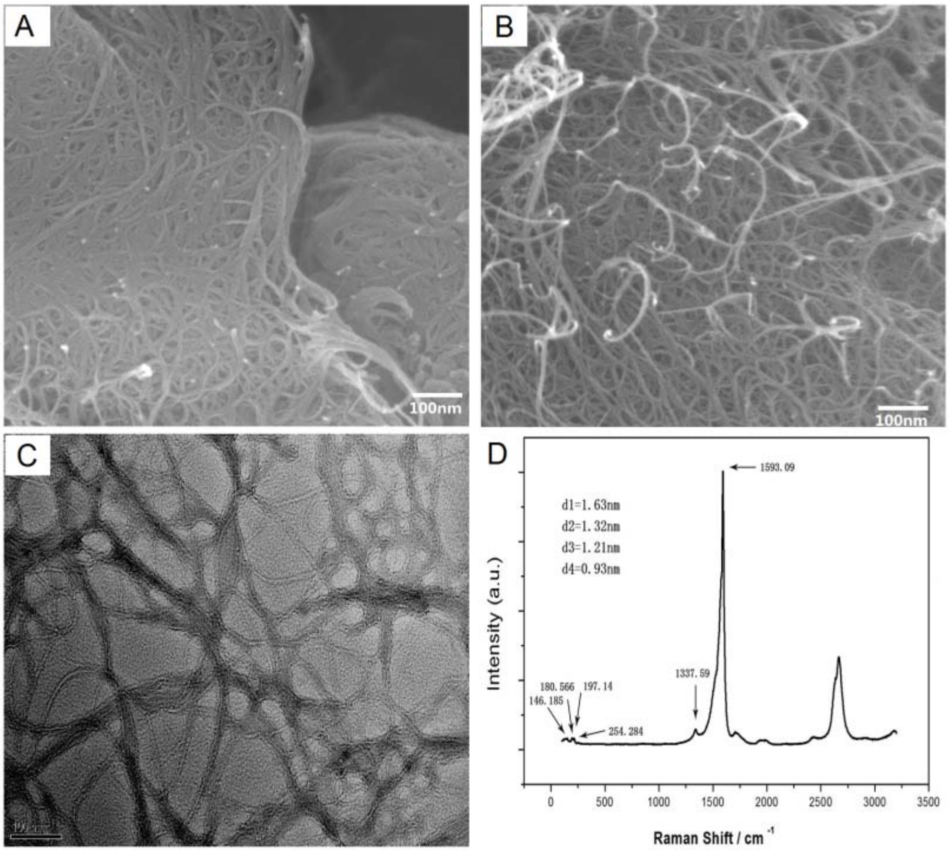

Figure S1. Characterization of SWCNTs. A, SEM image of SWCNTs suspended in distilled water; B, SEM image of SWCNTs suspended in 0.05% Tween-80 water solution; C, TEM image of SWCNTs; and D, Raman spectrum of SWCNTs at 633 nm excitation.

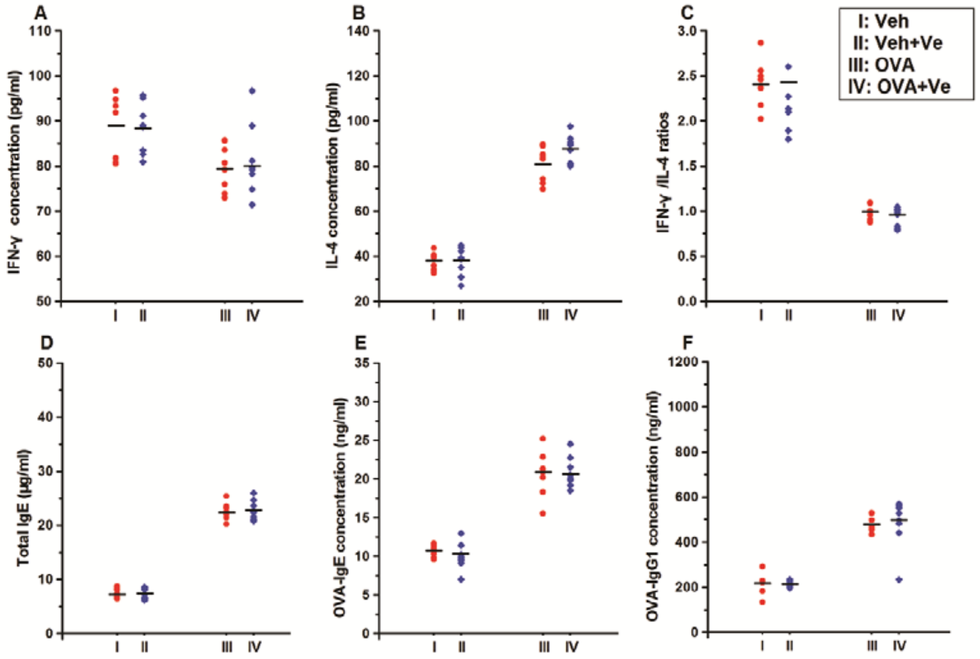

Figure S2. Effects of Ve on cytokine concentrations in lung tissue and serum Ig concentrations in Veh and OVA groups. Concentrations of IFN-γ, IL-4, IFN-γ/IL-4 ratios, tIg E, OVA-sIgE, and OVA-sIgG1 indicated in A–F, respectively.

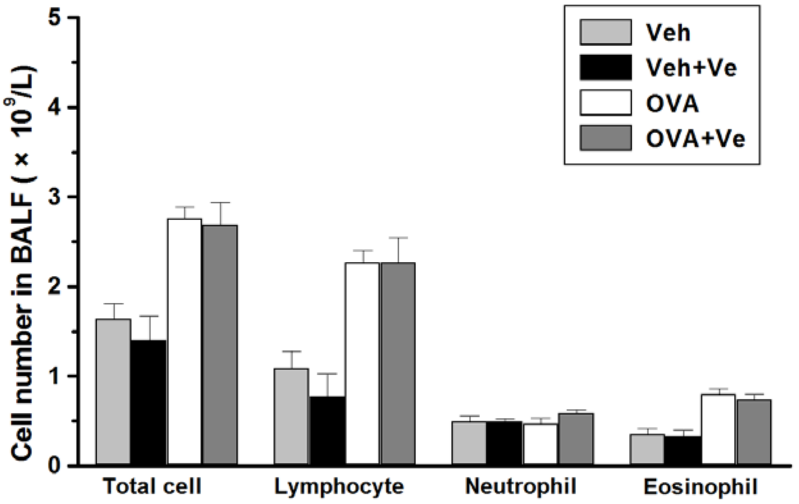

Figure S3. Effects of Ve on inflammatory cell recruitment in Veh and OVA groups.

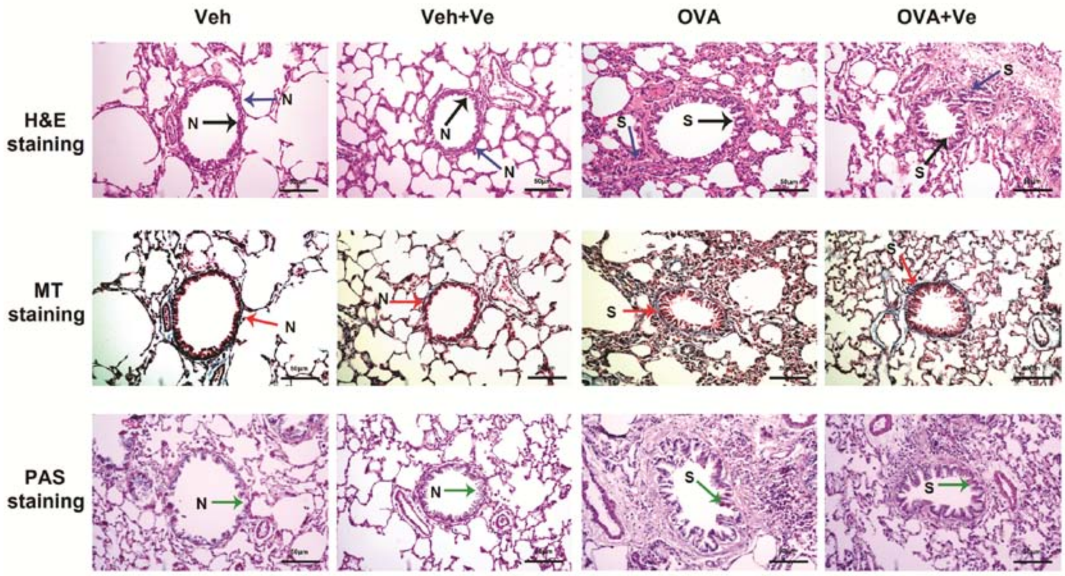

Figure S4. Effects of Ve visualized as histopathological changes in lung tissue from Veh and OVA groups. Black arrow, bronchial remodeling; blue arrow, lung tissue cell infiltration; red arrow, subepithelial collagen deposition (blue colored stain); green arrow, mucus hypersecretion (pale pink colored stain). N, S, M, and V indicate normal, slight, moderate, and severe changes, respectively.

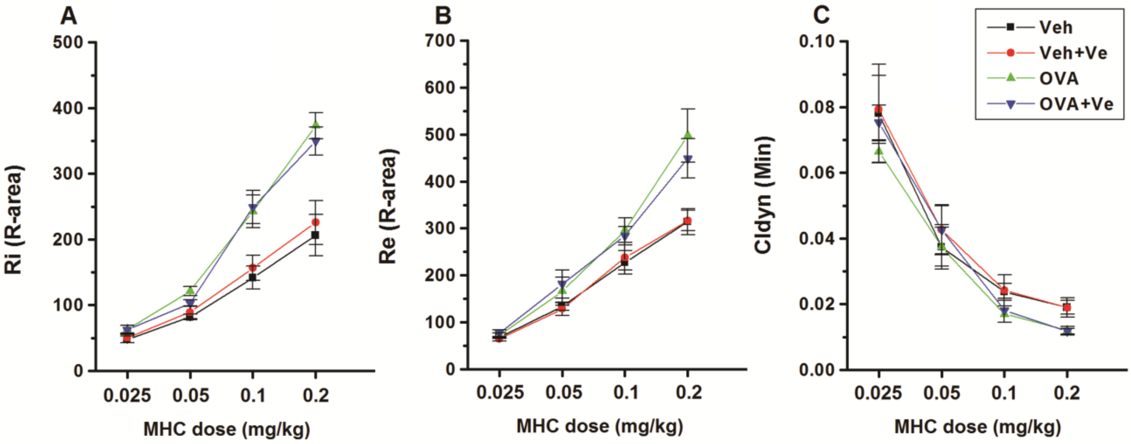

Figure S5. Effects of Ve on AHR in Veh and OVA groups. A–C, Ri, Re, and Cldyn values, respectively, of vehicle, vehicle+Ve, OVA, and OVA+Ve groups.

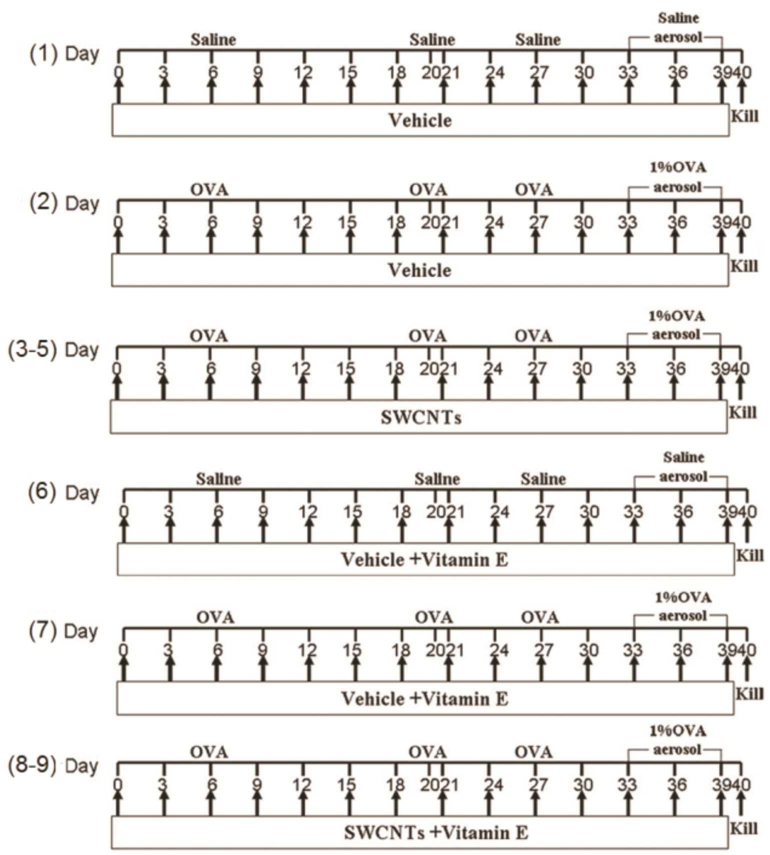

Figure S6. Study protocol.
